# Interference with Clp protease impairs carotenoid accumulation during tomato fruit ripening

**DOI:** 10.1093/jxb/erx491

**Published:** 2018-01-29

**Authors:** Lucio D’Andrea, Miguel Simon-Moya, Briardo Llorente, Ernesto Llamas, Mónica Marro, Pablo Loza-Alvarez, Li Li, Manuel Rodriguez-Concepcion

**Affiliations:** 1 Centre for Research in Agricultural Genomics (CRAG) CSIC-IRTA-UAB-UB, Campus UAB Bellaterra, Barcelona, Spain; 2 ICFO-Institut de Ciències Fotòniques, The Barcelona Institute of Science and Technology, Mediterranean Technology Park, Castelldefels, Barcelona, Spain; 3 Robert W. Holley Center for Agriculture and Health, USDA-ARS, Cornell University, Ithaca, NY, USA; 4 Plant Breeding and Genetics Section, School of Integrative Plant Science, Cornell University, Ithaca, NY, USA

**Keywords:** Carotenoid, chaperones, chromoplast, Clp protease, ClpB3, fruit, Orange, ripening, tomato

## Abstract

Profound metabolic and structural changes are required for fleshy green fruits to ripen and become colorful and tasty. In tomato (*Solanum lycopersicum*), fruit ripening involves the differentiation of chromoplasts, specialized plastids that accumulate carotenoid pigments such as β-carotene (pro-vitamin A) and lycopene. Here, we explored the role of the plastidial Clp protease in chromoplast development and carotenoid accumulation. Ripening-specific silencing of one of the subunits of the Clp proteolytic complex resulted in β-carotene-enriched fruits that appeared orange instead of red when ripe. Clp-defective fruit displayed aberrant chromoplasts and up-regulated expression of nuclear genes encoding the tomato homologs of Orange (OR) and ClpB3 chaperones, most probably to deal with misfolded and aggregated proteins that could not be degraded by the Clp protease. ClpB3 and OR chaperones protect the carotenoid biosynthetic enzymes deoxyxylulose 5-phosphate synthase and phytoene synthase, respectively, from degradation, whereas OR chaperones additionally promote chromoplast differentiation by preventing the degradation of carotenoids such as β-carotene. We conclude that the Clp protease contributes to the differentiation of chloroplasts into chromoplasts during tomato fruit ripening, acting in co-ordination with specific chaperones that alleviate protein folding stress, promote enzyme stability and accumulation, and prevent carotenoid degradation.

## Teaser

The stromal Clp protease complex influences chromoplast differentiation and ensures proper carotenoid metabolism during tomato fruit ripening, probably in co-ordination with plastidial chaperones.

## Introduction

Fruits are a major evolutionary innovation as they greatly contribute to the efficient dissemination of seeds. In particular, fleshy fruits undergo a ripening process after seed maturation that typically involves changes in organoleptic characteristics such as color, flavor, and texture. The resulting ripe fruit hence becomes an appealing food, eventually facilitating animal-assisted dispersal and germination of mature seeds (Seymour *et al.*, 2013; Llorente *et al.*, 2016*a*). One of the most studied models of fleshy fruit development is tomato (*Solanum lycopersicum*), an important crop worldwide. Upon fertilization, cell division and expansion result in a full-sized fruit with mature seeds but a green appearance due to the presence of chloroplasts. From this stage (referred to as mature green or MG), ripening causes chlorophyll breakdown and a dramatic increase in the production of carotenoid pigments that change the fruit color from green to orange (at the O stage) and eventually red (at the R stage) when fully ripe (Seymour *et al.*, 2013; Liu *et al.*, 2015; Llorente *et al.*, 2016*a*). At the structural level, chlorophyll loss and carotenoid overproduction during ripening are associated with the conversion of pre-existing chloroplasts into chromoplasts, which are plastids specialized in carotenoid storage (Li and Yuan, 2013; Pesaresi *et al.*, 2014).

**Fig. 1.**
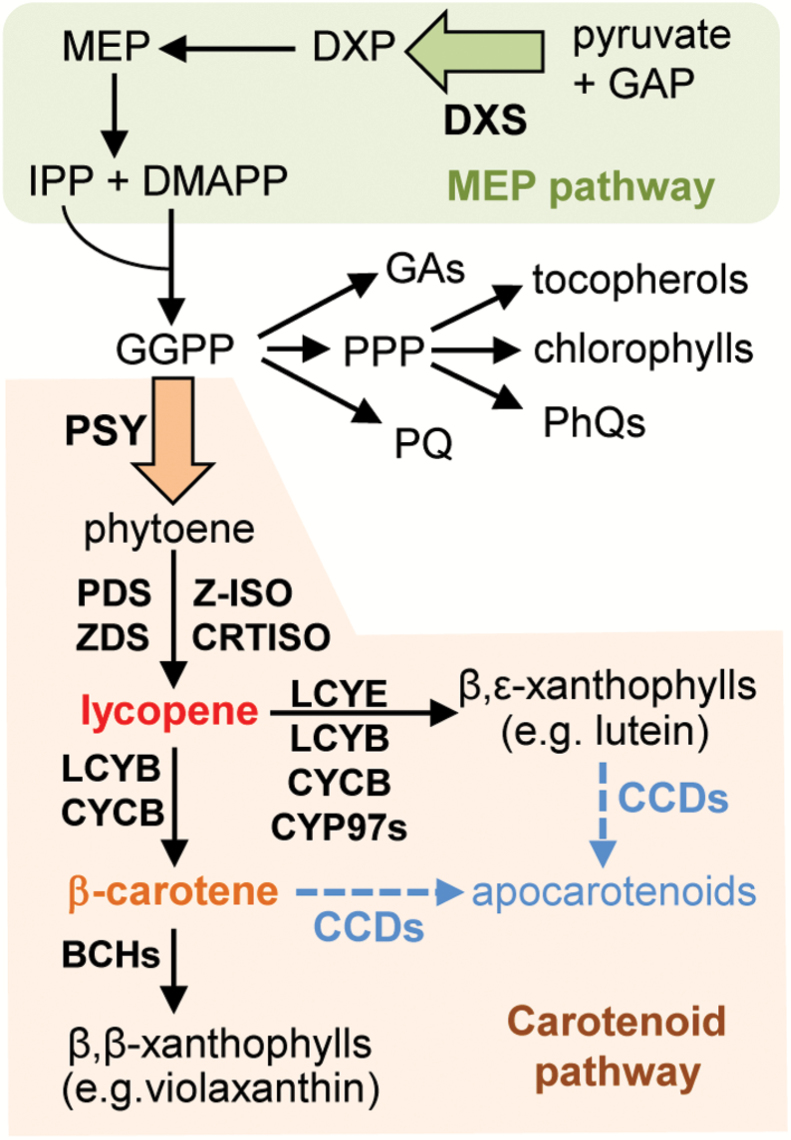
Carotenoid biosynthesis pathway. The methylerythritol 4–phosphate (MEP) pathway (green box) provides substrates for the production of several groups of plastidial isoprenoids, including carotenoids (orange box). Enzyme acronyms: DXS, deoxyxylulose 5-phosphate synthase; PSY, phytoene synthase; PDS, phytoene desaturase; ZDS, ζ-carotene desaturase; Z-ISO, 15-*cis*-ζ-carotene isomerase; CRTISO, carotenoid isomerase; LCYE, lycopene ε-cyclase; LCYB, lycopene β-cyclase; CYCB, chromoplast lycopene β-cyclase; CYP97s, cytochrome P450 carotenoid hydroxylases; BCHs, non-heme di-iron carotenoid hydroxylases; CCDs, carotenoid cleavage dioxygenases. Large arrows mark the rate-limiting steps catalyzed by DXS and PSY. GAP, glyceraldehyde 3-phosphate; DXP, deoxyxylulose 5-phosphate; IPP, isopentenyl diphosphate; DMAPP, dimethylallyl diphosphate; GGPP, geranylgeranyl diphosphate; GAs, gibberellins; PPP, phytyl diphosphate; PhQs, phylloquinones; PQ, plastoquinone.

Carotenoids are plastidial isoprenoids synthesized from metabolic precursors supplied by the methylerythritol 4-phosphate (MEP) pathway (Fig. 1). The initial step of the MEP pathway, catalyzed by deoxyxylulose 5-phosphate synthase (DXS), is limiting for carotenoid biosynthesis during tomato fruit ripening (Lois *et al.*, 2000; Enfissi *et al.*, 2005). The first committed step of the carotenoid pathway is the production of phytoene (a non-colored carotenoid) catalyzed by phytoene synthase (PSY). Metabolic control analysis demonstrated that, similar to that described for DXS in the MEP pathway (Wright *et al.*, 2014), PSY catalyzes the main flux-controlling step of the carotenoid pathway (Fraser *et al.*, 2002). After a series of desaturation and isomerization reactions, phytoene is converted into lycopene. Then, cyclization of the ends of the linear lycopene molecule with β or/and ɛ rings by lycopene cyclases produces carotenes such as pro-vitamin A β-carotene (β,β) and α-carotene (β,ɛ). Further oxidation of the rings results in the production of xanthophylls of β,β (e.g. violaxanthin) or β,ɛ (e.g. lutein) type (Fig. 1; Supplementary Table 1Supplementary Table S1 at *JXB* online). Carotenoid metabolism involves their degradation by either non-enzymatic or enzyme-mediated cleavage, giving rise to a variety of metabolites collectively referred to as apocarotenoids (Fig. 1). They include hormones (such as abscisic acid and strigolactones) and volatiles that determine the characteristic aroma of ripe fruit (Simkin *et al.*, 2004; Wei *et al.*, 2016; Tieman *et al.*, 2017).

Chloroplasts of MG fruit mainly accumulate β-carotene and xanthophylls. During ripening, however, carotenoid biosynthesis is boosted and lycopene progressively accumulates in chromoplasts to provide eventually the characteristic red color of ripe fruit (Liu *et al.*, 2015; Llorente *et al.*, 2016*a*). The changes in the quantitative and qualitative carotenoid profiles of tomato fruits that occur during ripening are generally assumed to be mainly orchestrated at the gene expression level (Seymour *et al.*, 2013; Liu *et al.*, 2015; Giovannoni *et al.*, 2017). Thus, the expression of genes encoding the specific DXS and PSY isoforms that contribute to carotenoid biosynthesis in tomato fruit (*DXS1* and *PSY1*, respectively) co-ordinately increases during ripening (Lois *et al.*, 2000), resulting in a strong activation of the metabolic flux to the carotenoid pathway. Furthermore, down-regulation of genes encoding lycopene cyclases as the fruit ripens results in the progressive enrichment in lycopene (Pecker *et al.*, 1996; Ronen *et al.*, 1999). While the existence of regulatory mechanisms beyond the control of gene expression is widely acknowledged (Fraser *et al.*, 2009), their molecular nature remains little studied.

Work in the model plant *Arabidopsis thaliana* has shown that the control of protein turnover has a major impact on the levels and activity of DXS and PSY enzymes (Flores-Pérez *et al.*, 2008; Pulido *et al.*, 2013; Yuan *et al.*, 2015; Zhou *et al.*, 2015; Perello *et al.*, 2016; Pulido *et al.*, 2016; Llamas *et al.*, 2017). The stromal Clp protease complex degrades DXS enzymes that become inactive and aggregate, whereas the Hsp100-type chaperone ClpB3 promotes their solubility and hence enzymatic activity (Perello *et al.*, 2016; Pulido *et al.*, 2016; Llamas *et al.*, 2017). PSY activity and stability are promoted by interaction with Orange (OR) proteins, DnaJ-like chaperones that independently act as positive regulators of chromoplast differentiation and repressors of carotenoid degradation (Zhou *et al.*, 2015; Park *et al.*, 2016; Chayut *et al.*, 2017). Here we explored whether the Clp protease complex also had a role in the post-transcriptional regulation of carotenoid accumulation during tomato ripening. By reducing Clp protease activity in ripening fruit, we unveiled a role for this proteolytic complex in carotenoid biosynthesis and accumulation, most probably in co-ordination with tomato ClpB3 and OR chaperones.

## Materials and methods

### Plant material and growth conditions

Tomato (*Solanum lycopersicum*) plants of the Micro-Tom (MT) variety were used for experiments. Plants were grown under standard greenhouse conditions (14 h light at 27 ± 1 °C and 10 h dark at 22 ± 1 °C). Fruit stages were established according to the days post-anthesis (DPA) and verified based on the color of the fruit in untransformed MT plants. Briefly, independent tomato flowers were tagged at the anthesis stage and fruit material was collected at 36 DPA (MG stage), 47 DPA (O stage), and 52 DPA (R stage). For heat shock treatments, fruits at the MG stage were harvested from the plant and incubated in a growth chamber at 37 ºC in the light for 4 d. Then, they were incubated at 25 ºC for a further 10 d. Control fruits were incubated at 25 ºC in the light for the whole 2 week period.

### Phylogenetic analysis

Sequences for Arabidopsis Clp complex subunits, chaperones, and proteins involved in carotenoid metabolism were used as queries without their plastid-targeting peptides (predicted using ChloroP) to search for putative homologs in tomato and other plants using BLAST on two different sequence databases: the Solanaceae Genomics Network (http://solgenomics.net/) and the National Center for Biotechnology Information (www.ncbi.nlm.nih.gov/). Accessions of the identified proteins are indicated in Supplementary Table 1Supplementary Tables S1 and Supplementary Table 1S2. Protein alignments were performed using MUSCLE (Edgar, 2004), and an unrooted tree was constructed using MEGA6 as described (Hall, 2013).

### Constructs for transient and stable expression in plants

An artificial miRNA (amiRNA) targeting the *ClpR1* gene was designed following the recommendations available on the Web MicroRNA Designer (WMD3) online tool (http://wmd3.weigelworld.org/cgi-bin/webapp.cgi). Plasmid pRS300 was used as template to introduce into the *miR319a* precursor by site-directed mutagenesis (Schwab *et al.*, 2006; Ossowski *et al.*, 2008). The overlapping PCR amplification steps were carried out as described (Fernandez *et al.*, 2009) using appropriate primers (Supplementary Table 1Supplementary Table S3), and the resulting products were cloned into pDONR221P4r-P3r. The generated constructs were named pEF4r-amiR1_1-3r and pEF4r-amiR1_2-3r. The pEF4r-amiC-3r plasmid was constructed after generating the amiC sequence by site-directed mutagenesis PCR using the amiR1_1 sequence as a template. The three constructs were then recombined with plasmids pEF1-2x35S-4 and pEF3-Tnos-2 (Estornell *et al.*, 2009), and the resulting triple recombination was subcloned into the binary vector pKGW,0 for transient expression assays in tomato leaves, as described (Llorente *et al.*, 2016*b*). For stable expression experiments, constructs harboring the corresponding amiRNA sequences under the transcriptional control of the ripening-specific *E8* promoter were generated by a similar triple recombination using plasmid pEF1-E8-4 instead of pEF1-2x35S-4 (Estornell *et al.*, 2009). Transformation of tomato MT plants was carried out as described (Llorente *et al.*, 2016*b*).

### Transcript, protein, and metabolite analysis

Tomato fruit pericarp samples were frozen in liquid nitrogen immediately after collection and then lyophilized. The generated material was then used for the analysis of gene expression and metabolite profiles. Total RNA was isolated and used for quantitative RT-PCR (qPCR) analysis of transcript levels as described (Llorente *et al.*, 2016*b*) using the primers listed in Supplementary Table 1Supplementary Table S3. Normalized transcript abundance was calculated using the tomato *ACT* gene (Solyc04g011500) as a reference (Llorente *et al.*, 2016*b*). This gene was selected based on its stable expression during fruit ripening (Supplementary Table 1Supplementary Fig. S1) and expression levels more similar to those of carotenoid-related genes compared with other highly stable normalizers (Supplementary Table 1Supplementary Table S4). Carotenoids were extracted as described (Llorente *et al.*, 2016*b*) and their qualitative and quantitative profiles were determined by HPLC (Supplementary Table 1Supplementary Fig. S2). For protein analyses, lyophilization was not performed and frozen samples were directly used for protein extraction and immunoblot analyses as described (Pulido *et al.*, 2013), using antibodies against Arabidopsis DXS (Pulido *et al.*, 2013) and PSY (Maass *et al.*, 2009) proteins.

### Raman and transmission electron microscopy

For Raman spectral imaging, fresh fruit pericarp sections were cut into 300 μm thick sections using a vibratome. Sections kept in water (to avoid dehydration) were then observed with a Renishaw inVia confocal Raman microscope equipped with a ×60 water immersion objective (NA=1, Nikon) and a 532 nm laser using a 2.7 s integration time, 20 mW power, and 0.6 μm pixel size. Data from Raman maps were processed using the Multivariate Curve Resolution (MCR) algorithm (Jaumot *et al.*, 2005; Piqueras *et al.*, 2011) by means of the PLS Toolbox (Eigenvector Research) and Matlab. For TEM, fruit pericarp samples were cut into small pieces and immediately prepared as described (Flores-Pérez *et al.*, 2008). Specimens were observed using a FEI Tecnai G2 F20 electron microscope operating at 200 keV and recorded using a FEI Eagle 4 k×4 k CCD camera.

### Statistical analyses

Student’s *t*-test, ANOVA followed by Newman–Keuls multiple comparison post-hoc test, and Pearson correlation coefficients (*r* values) were calculated using GraphPad Prism 5.0a (GraphPad Software).

## Results and Discussion

### Genes encoding Clp protease subunits are induced during tomato fruit ripening

**Fig. 2.**
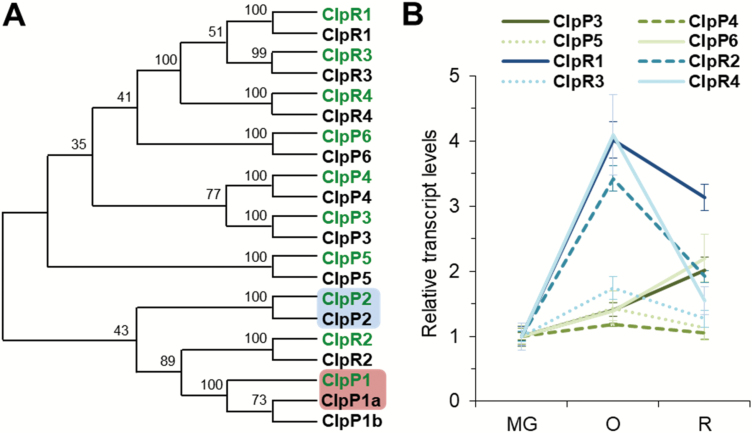
Tomato Clp subunits are up-regulated during fruit ripening. (A) Maximum Likelihood tree constructed with Arabidopsis (green) and tomato (black) protein sequences. See Supplementary Table S2 for accessions. Mitochondrial subunits are boxed in blue, and plastome-encoded subunits are boxed in red. (B) Quantitative RT-PCR (qPCR) analysis of transcript levels for nuclear-encoded Clp protease subunits in Micro-Tom (MT) fruit at the indicated ripening stages. Data are represented relative to the levels in MG fruit and they correspond to the mean ±SE from *n* ≥3 independent fruits.

The Clp proteolytic complex is found in all plastid types, including chromoplasts, where it is expected to have a similar subunit composition to that reported in the chloroplasts of Arabidopsis and other plants (Peltier *et al.*, 2004). Some proteins with similarity to Clp complex subunits have actually been found in tomato fruit (Barsan *et al.*, 2012). To have a complete view of the genes and proteins forming the Clp protease in tomato, we searched the tomato genome for the different subunits of the complex (Supplementary Table 1Supplementary Fig. S1). In Arabidopsis, the proteolytic core of the complex is formed by two heptameric rings of plastome-encoded ClpP1 and nuclear-encoded ClpR1-R4 and ClpP3-P6 proteins stabilized by plant-specific ClpT1-T2 subunits. A dynamically interacting hexameric ring of Hsp100 chaperones (ClpC1-C2 and ClpD) unfolds protein substrates for translocation into the proteolytic chamber (Peltier *et al.*, 2004; Nishimura and van Wijk, 2015). Using the Arabidopsis proteins as queries, we identified tomato homologs for all these subunits (Fig. 2; Supplementary Table 1Supplementary Table S2). Only in the case of ClpP1 did we find more than one potential homolog, tentatively named ClpP1a and ClpP1b (Fig. 2A). Two identical copies of ClpP1a were retrieved from the tomato plastid and nuclear genomes available at the Solanaceae Genomics Network database. However, the presumably chromosomal copy (Solyc01g007490) is flanked by plastome sequences, suggesting that this might be an assembly artifact and that it actually corresponds to the single plastome gene encoding ClpP1a. In the case of ClpP1b (Solyc09g065790), algorithms such as TargetP and ChloroP failed to detect a targeting signal (Supplementary Table 1Supplementary Table S2). The ClpP1b protein also lacks two of the three conserved residues of the catalytic triad (Supplementary Table 1Supplementary Fig. S3), suggesting that it might not be a stromal Clp protease complex subunit. The rest of the tomato homologs encoding subunits of the proteolytic core and the associated chaperones were predicted to harbor plastid-targeting peptides, with the exception of ClpP2, the only family member that is targeted to mitochondria to form the Clp protease of this organelle (Fig. 2A; Supplementary Table 1Supplementary Table S2).

As shown in Fig. 2B and Supplementary Table 1Supplementary Fig. S1, the identified genes were up-regulated during fruit ripening. Most were highly induced during the transition from breaker to O fruit, when chloroplasts lose their photosynthetic structures and differentiate into carotenoid-accumulating chromoplasts. The *Clp1b* gene, however, was found not to be up-regulated during fruit ripening (Supplementary Table 1Supplementary Fig. S1), further supporting the conclusion that it does not encode a Clp protease subunit. The observed up-regulated expression of the complex subunits during tomato fruit ripening suggests that increasing Clp protease activity might be instrumental for this developmental process.

### Ripening-specific silencing of the tomato *ClpR1* gene results in orange ripe fruits

Down-regulation of individual Clp complex subunits in Arabidopsis mutants typically results in lower proteolytic activity of the whole complex, and the same might be expected to happen in tomato fruit (Nishimura and van Wijk, 2015). To down-regulate Clp protease activity in tomato, we chose to silence the gene encoding ClpR1 based on the well-known and non-lethal phenotype of Arabidopsis ClpR1-defective mutants (Koussevitzky *et al.*, 2007; Flores-Pérez *et al.*, 2008; Kim *et al.*, 2009). Silencing was achieved using two amiRNAs designed to target *ClpR1*-specific sequences (Supplementary Table 1Supplementary Fig. S4). As a control, an inactive amiRNA was generated by introducing critical mismatches in the so-called seed region of one of the target sequences (Supplementary Table 1Supplementary Fig. S4). The presumably active (*amiR1_1* and *amiR1_2*) and inactive (*amiC*) amiRNA versions were initially cloned for transient expression in tomato leaves. After agroinfiltration and qPCR analysis of *ClpR1* transcript levels, it was confirmed that both *amiR1_1* and *amiR1_2* were effective in decreasing the accumulation of *ClpR1* transcripts (Supplementary Table 1Supplementary Fig. S4). In contrast, the *amiC* construct did not trigger *ClpR1* silencing in tomato cells. Based on the described results, *amiR1_1* (from here on, *amiR1*) and *amiC* (as a negative control) were selected to be stably expressed in tomato.

**Fig. 3.**
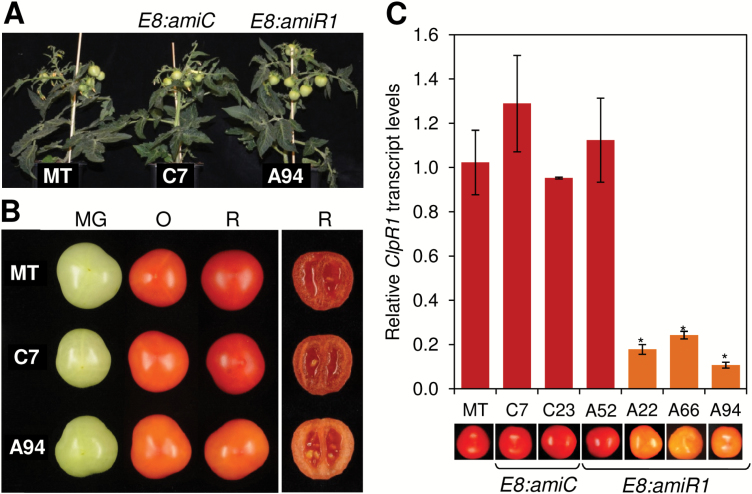
Reduced *ClpR1* transcript levels result in orange ripe fruits. (A) Representative greenhouse-grown plants either untransformed (MT) or transformed with *E8:amiC* (line C7) or *E8:amiR1* (line A94) constructs. Transgenic plants of the T_2_ generation are shown. (B) Representative fruits of the indicated lines at three developmental stages (MG, O, and R). (C) qPCR analysis of *ClpR1* transcript levels in ripe fruits from MT plants or T_2_ generation *E8:amiC* (C7 and C23) and *E8:amiR1* (A52, A22, A66, and A94) transgenic lines. Values are shown relative to those in MT fruit and correspond to the mean ±SE (*n* ≥3). Asterisks mark statistically significant differences relative to MT samples (*P*<0.05). Representative fruits of every line at the R stage are also shown.

To analyze the role of the Clp protease complex specifically in tomato fruit, we chose to express the *amiR1* construct under the control of the ripening-induced *E8* promoter (Estornell *et al.*, 2009). Similar to what was observed for most genes encoding Clp protease complex subunits (Fig. 2B; Supplementary Table 1Supplementary Fig. S1), *E8* expression remains low in immature, MG, and breaker fruit, but strongly increases at the O stage and remains high in R fruit (Supplementary Table 1Supplementary Fig. S5A, Supplementary Table 1B). We therefore specifically expected to decrease Clp protease activity when most critical during ripening. Following transformation of tomato plants of the MT variety, we selected 10 independent T_1_ plants transformed with the active *amiR1* construct (*E8:amiR1* or A lines) and seven more carrying the inactive *amiC* sequence (*E8:amiC* or C lines). Among them, four *E8:amiR1* lines (A22, A52, A66, and A94) and two *E8:amiC* lines (C7 and C23) were taken to the next generation for further analyses (Fig. 3).

As expected from the fruit ripening-specific profile of *E8* promoter activity, transgenic T_2_ plants were indistinguishable from untransformed MT controls grown in the same greenhouse room (Fig. 3A). All the lines developed normal MG fruits at ~36 DPA (Fig. 3A–B) and they also reached the breaker stage at the same time, ~41 DPA (Supplementary Table 1Supplementary Fig. S6). Fruits at the O stage (47 DPA) also appeared similar in all lines, but visual differences were found in ripe (R, 52 DPA) fruits (Fig. 3B). Again, this can be expected as the *E8* promoter is most active at the O stage (Estornell *et al.*, 2009), and hence the silencing effects of the amiRNA construct are most obvious at later stages of ripening (Supplementary Table 1Supplementary Fig. S5C). A wild-type phenotype of normal red ripe fruits was observed in all *E8:amiC* lines and also in *E8:amiR1* line A52 (Fig. 3C). However, lines A22, A66, and A94 developed fruits that retained an orange coloration at the R stage (Fig. 3C). Analysis of *ClpR1* transcript levels in R fruits from lines C7, C23, and A52 (i.e. those developing red ripe fruits) showed no differences from the MT control (Fig. 3C). In contrast, the orange-colored R fruits from lines A22, A66, and A94 showed strongly reduced levels of *ClpR1* transcripts (Fig. 3C). Consistent with the expected activity of the *E8* promoter, *ClpR1* silencing was hardly detected at the O stage (Supplementary Table 1Supplementary Fig. S5C).

We next tested whether the abnormal orange coloration of *ClpR1*-silenced ripe fruit was due to a general arrest of ripening at the O stage. The actual fruit developmental stage was assessed by analyzing the expression of marker genes involved in ethylene synthesis and response (*ACS2* and *E8*) and cell softening (*PG2A*). In wild-type fruits, the expression of these three genes is highest at the O stage and then dramatically drops in R fruit (Supplementary Table 1Supplementary Fig. S5A). If the orange phenotype of *ClpR1*-silenced ripe fruit was the consequence of a general fruit ripening arrest at the O stage, it would be expected that the transcript levels of marker genes in A94 fruit at the R stage were similar to those in MT fruit at the O stage (i.e. much higher than those in ripe MT fruit). However, very similar transcript levels were detected for all these genes in control MT (red) and silenced A94 (orange) fruits collected at 52 DPA (Supplementary Table 1Supplementary Fig. S5D). These results, together with the observation that A94 fruit retained their orange coloration when over-ripe (up to 80 DPA), suggest that the orange phenotype displayed by ripe fruits with reduced Clp protease activity is not due to a general blockage of the ripening program but must be caused by alterations in specific Clp-regulated processes related to fruit pigmentation.

### Ripe *E8:amiR1* fruit are enriched in the pro-vitamin A carotenoid β-carotene

**Fig. 4.**
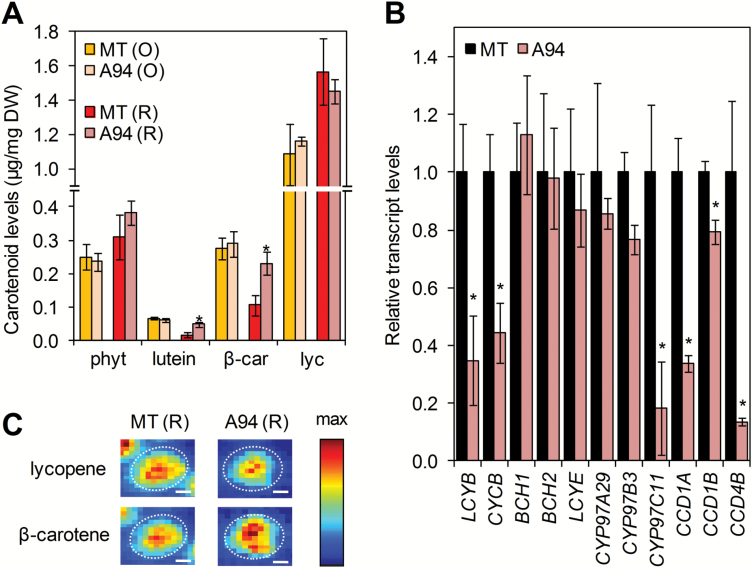
*ClpR1*-silenced fruits are enriched in β-carotene. (A) Levels of individual carotenoids in fruits of the indicated lines at O and R stages (phyt, phytoene; β-car, β-carotene; lyc, lycopene). Data correspond to the mean ±SE of *n*=3 independent fruit. (B) qPCR analysis of the levels of transcripts from the indicated genes in ripe MT and A94 fruits. See Fig. 1 and Supplementary Table 1Supplementary Table S1 for accessions. Means ±SE of *n* ≥5 fruits are shown relative to MT samples. Asterisks mark statistically significant differences relative to reference MT samples (*P*<0.05). (C) Raman imaging of lycopene and β-carotene accumulation in individual plastids from ripe MT and A94 fruits. Chromoplast perimeters are indicated with dashed white lines. Colors represent the MCR scores (i.e. relative abundance) of the corresponding pure components according to the scale shown in the box. Scale bars correspond to 2 µm.

The T_3_ generation of transgenic plants displayed the same ripe fruit phenotypes described above, indicating that this is a stable feature. To investigate the metabolic basis of the coloration of Clp-silenced ripe fruit, the carotenoid profile of the pericarp of MT and A94 fruit at the O and R stages (i.e. 47 and 52 DPA, respectively) was compared (Fig. 4). MT and transgenic A94 lines showed similar levels of the most abundant carotenoids in O fruit (i.e. lycopene, β-carotene, phytoene, and lutein). As ripening continued, levels of β-carotene (orange) and lutein (yellow) decreased and lycopene (red) increased in MT fruit (Fig. 4A), hence changing the fruit color from orange (at the O stage) to red (at the R stage). In contrast, levels of β-carotene and lutein hardly changed when A94 fruit reached the R stage (Fig. 4A), which might explain the orange color of ClpR1-defective ripe fruit (Fig. 3). Due to lack of major changes in lycopene, the most abundant carotenoid in ripe fruit (Supplementary Table 1Supplementary Fig S2), the amount of total carotenoids remained similar in the R fruit of MT (2.00 ± 0.25 µg mg^–1^ DW) and A94 (2.12 ± 0.09 µg mg^–1^ DW).

Analysis of the expression of genes related to β-carotene and lutein biosynthesis and degradation by qPCR showed reduced levels of transcripts for lycopene β-cyclases (LCYB and CYCB), carotenoid ε-hydroxylase (CYP97C11), and carotenoid cleavage enzymes (CCDs) in ripe A94 fruit compared with MT controls (Fig. 4B). Cyclases transform lycopene into β-carotene (β-cyclases) or α-carotene (both β- and ε-cyclases), which are next oxygenated to xanthophylls by specific hydroxylases (Fig. 1). In particular, CYP97C11 acts on the ε-ring of α-carotene to produce lutein (Stigliani *et al.*, 2011). From all CCDs present in tomato, only transcripts encoding isoforms CCD1A, CCD1B, and CCD4B (Simkin *et al.*, 2004; Wei *et al.*, 2016) were detected in R fruit at reliable levels by qPCR. All three of them were significantly reduced in A94 samples compared with MT controls (Fig. 4B). Assuming that reduced levels of transcripts result in lower levels of protein and eventually decreased enzymatic activity, down-regulation of transcripts for LCYB, CYCB, and CYP97C11 in Clp-defective fruit suggests that the production of β-carotene and lutein might be repressed in the transgenic fruit, whereas decreased levels of CCDs would prevent their degradation. An altered balance between biosynthesis and degradation might eventually contribute to the enhanced accumulation of β-carotene and lutein detected in Clp protease-defective ripe fruits (Fig. 4A). These results, however, do not exclude other mechanisms (including those independent of changes in gene expression) whose impairment in A94 fruit might contribute to its metabolic and visual phenotype.

### Clp-defective fruits contain atypical chromoplasts

**Fig. 5.**
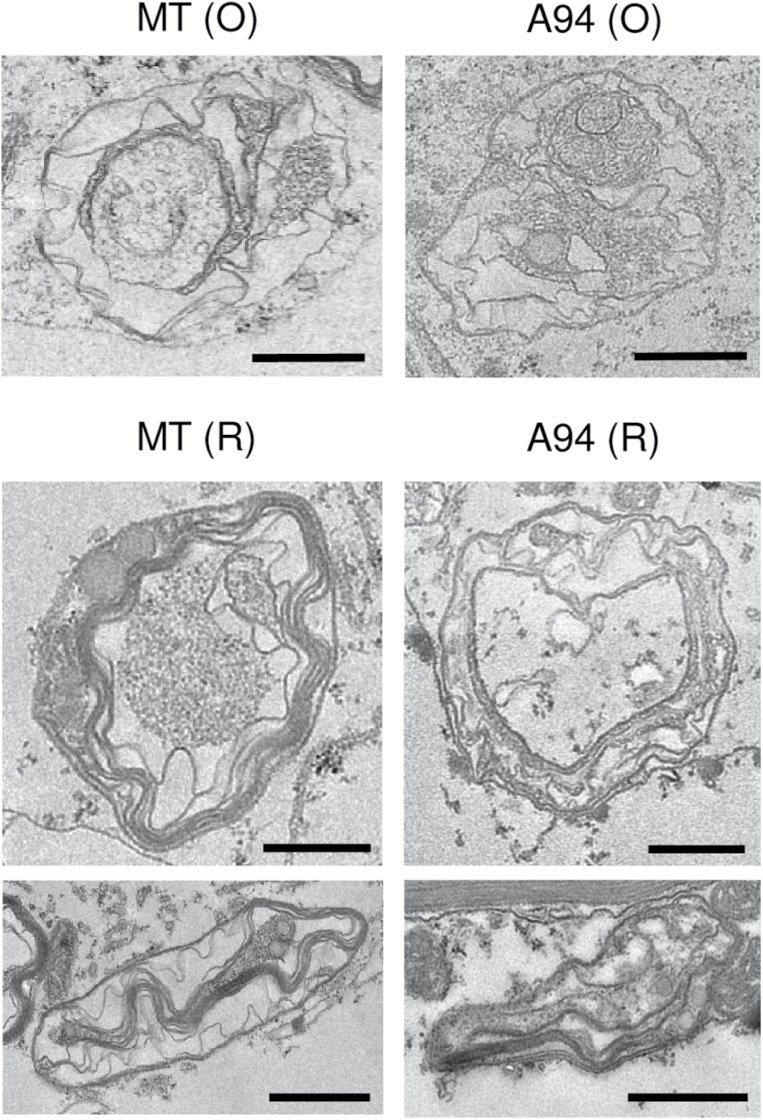
Clp-defective ripe fruits contain atypical chromoplast types. TEM images of representative chromoplasts from MT and A94 fruits at O and R stages. Scale bars correspond to 1 µm.

Clp protease is essential for chloroplast development in Arabidopsis (Nishimura and van Wijk, 2015). Because chromoplast differentiation involves major changes in plastid protein contents (Barsan *et al.*, 2012; Suzuki *et al.*, 2015; Zeng *et al.*, 2015) and the genes encoding Clp protease complex subunits are up-regulated during tomato fruit ripening (Fig. 2B; Supplementary Table 1Supplementary Fig. S1), we reasoned that Clp activity might be important for the proper differentiation of chloroplasts (rich in β-carotene and lutein) into chromoplasts. To test this hypothesis, fruits at the O and R stages were collected from MT and A94 lines and analyzed by TEM observation of plastid ultrastructure (Fig. 5).

For consistency, we focused on the cells of the exocarp (i.e. the most external layer of the pericarp) in the boundary with the mesocarp. As expected, chromoplasts from MT and A94 fruits were very similar at the O stage (Fig. 5). At the R stage, MT fruits displayed a fairly homogeneous population of typical globular/crystalloid chromoplasts (Fig. 5). These chromoplasts contained large vesicles and plastoglobules together with the membrane remnants of lycopene crystals; that is, the envelopes that shrink into an undulating shape when crystals are lost during the dehydration step of the TEM sample preparation procedure (Fig. 5). In contrast, most chromoplasts in R fruit from A94 plants contained disorganized membranes with very few globular structures (Fig. 5). These observations confirmed aberrant chromoplast development in Clp-defective fruit, coincident with the abnormal accumulation of carotenoids that led to the visual phenotype (orange ripe fruit).

### Clp-defective tomato fruits show increased expression of chaperone-encoding genes and higher levels of PSY and DXS enzymes

**Fig. 6.**
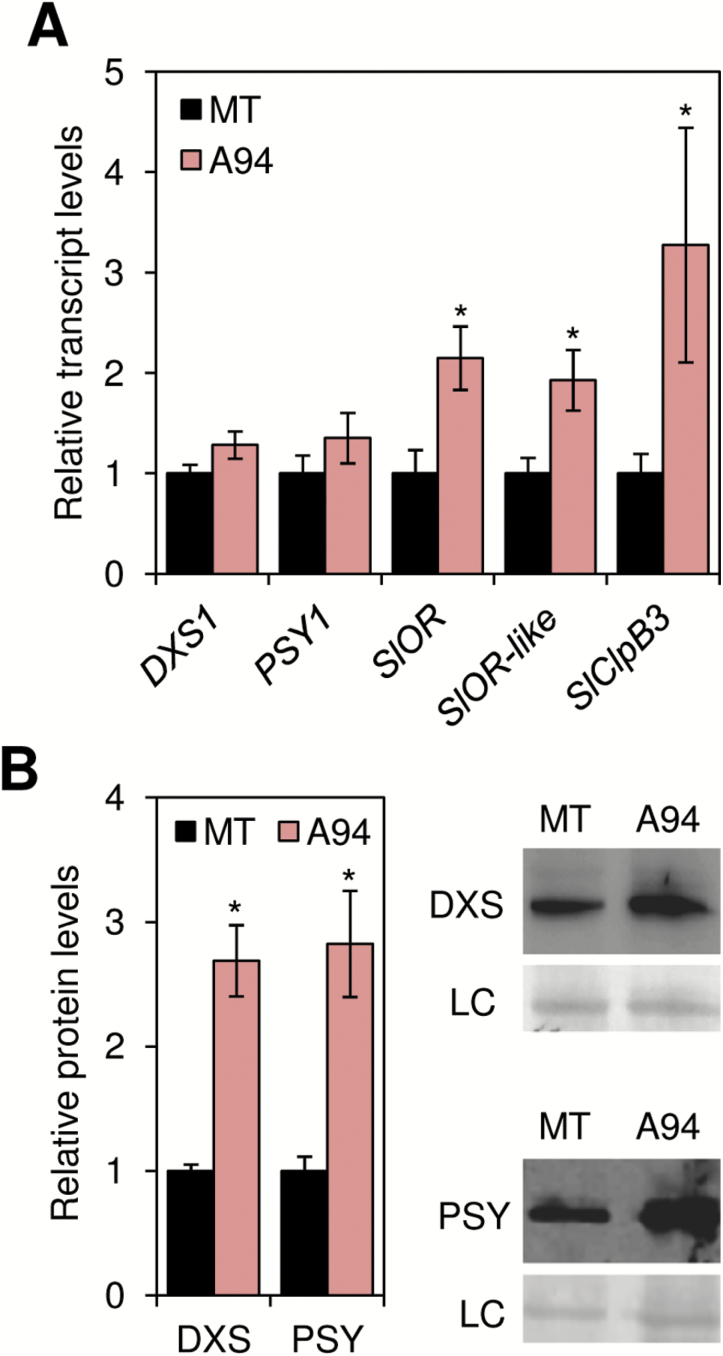
Reduced Clp activity up-regulates chaperone-encoding genes and promotes the accumulation of DXS and PSY enzymes. (A) qPCR analysis of transcript levels for the indicated genes in ripe fruit from MT and A94 plants. Data correspond to the mean and SE values from *n* ≥3 independent fruits and are presented relative to MT fruit. Asterisks mark statistically significant differences relative to MT samples (*P*<0.05). (B) Immunoblot analyses of DXS and PSY proteins in ripe MT and A94 fruits. Representative blots are shown on the right (LC, loading control). The plot shows the quantification of protein levels (mean ±SE) from *n* ≥4 fruits, represented relative to MT samples. Asterisks mark statistically significant differences relative to MT samples (*P*<0.05).

Chromoplast structures are often linked to the type of carotenoids that they store (Nogueira *et al.*, 2013; Lado *et al.*, 2015; Suzuki *et al.*, 2015; Zeng *et al.*, 2015). It is therefore likely that the atypical ultrastructure of chromoplasts developed in *ClpR1*-silenced R fruit was associated with their altered carotenoid content. Indeed, Raman spectral imaging of the distribution of lycopene and β-carotene in chromoplasts of ripe MT and A94 fruit confirmed a higher proportion of β-carotene in transgenic samples (Fig. 4C). Chromoplasts similar to those found in ripe A94 fruit have been previously observed in other plant systems (such as cauliflower curds and Arabidopsis calli) in which β-carotene overaccumulation was triggered by overexpression of genes encoding OR chaperones (Yuan *et al.*, 2015; Park *et al.*, 2016). As shown in Supplementary Table 1Supplementary Table S1, we identified two genes encoding OR proteins in tomato, here referred to as SlOR (Solyc03g093830) and SlOR-like (Solyc09g010110) based on their similarity to the Arabidopsis proteins (Supplementary Table 1Supplementary Fig. S7). Both genes are induced in the transition from breaker to O and then down-regulated when fruits reach the R stage (Supplementary Table 1Supplementary Fig. S8), a pattern of expression very similar to that observed for most Clp protease subunit genes (Supplementary Table 1Supplementary Fig. S1). Most interestingly, transcript levels for the tomato SlOR and SlOR-like isoforms were up-regulated ~2-fold in ripe A94 fruit compared with MT controls (Fig. 6). Work in different plant systems has shown that direct binding of both OR and OR-like proteins to PSY renders the enzyme more stable and catalytically active, possibly by achieving correct folding and/or preventing proteolytic degradation (Zhou *et al.*, 2015; Park *et al.*, 2016). Consistent with the conclusion that increased OR activity resulting from enhanced gene expression in Clp protease-defective A94 fruits (Fig. 6A) could also promote PSY stability in tomato, immunoblot analysis of PSY accumulation in ripe A94 and MT fruit showed higher enzyme levels in transgenic fruit (Fig. 6B), despite similar levels of *PSY1* transcripts (Fig. 6A).

Constitutive or induced down-regulation of Clp protease activity in chloroplasts has been previously shown to cause an enhanced accumulation of plastidial chaperones, most probably to deal with general protein folding stress caused by reduced protein degradation (Nishimura and van Wijk, 2015; Rochaix and Ramundo, 2015; Llamas *et al.*, 2017). OR or OR-like proteins do not overaccumulate in Clp-defective chloroplasts, perhaps because these chaperones do not have a relevant role in this plastid type. In contrast, the levels of the ClpB3 chaperone have been shown to increase dramatically when Clp activity is compromised (Ramundo *et al.*, 2014; Nishimura and van Wijk, 2015; Llamas *et al.*, 2017). ClpB3 is encoded by a single gene in tomato (*SlClpB3*, Solyc02g088610) (Supplementary Table 1Supplementary Table S1) (Mishra and Grover 2016), showing a pattern of expression during fruit ripening similar to that of Clp protease subunit genes (Supplementary Table 1Supplementary Fig. S1) but also *SlOR*, *SlOR-like*, *PSY1*, and *DXS1* (Supplementary Table 1Supplementary Fig. S8). Also similar to *SlOR* and *SlOR-like*, *SlClpB3* transcript levels were higher in ripe A94 fruit compared with untransformed controls (Fig. 6A). This result confirms that defects in Clp protease activity during tomato fruit ripening eventually result in up-regulated expression of nuclear genes encoding plastidial chaperones. Similarly, work in Arabidopsis has shown that saturation of the Clp protease capacity and aggregation of misfolded proteins can unleash a retrograde signaling pathway to induce the expression of chromosomal genes for chloroplast-targeted chaperones such as ClpB3 (Llamas *et al.*, 2017). As a consequence, folding capacity is increased to restore protein homeostasis in the chloroplast. In particular, ClpB3 promotes correct folding and hence prevents Clp-mediated degradation of DXS (Perello *et al.*, 2016; Pulido *et al.*, 2016; Llamas *et al.*, 2017). The same appears to be true in tomato, as levels of DXS protein were higher in Clp-defective fruit (Fig. 6B) despite the fact that no difference from the MT control was found at the level of transcripts (Fig. 6A).

**Fig. 7.**
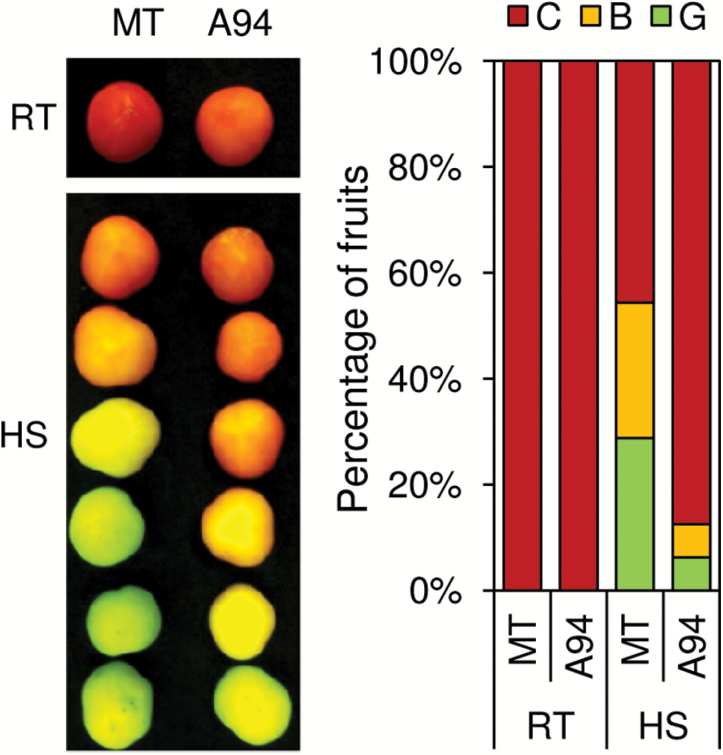
Defective Clp activity improves fruit tolerance to heat stress. Fruits from MT and A94 plants were harvested at the MG stage and then incubated in a growth chamber at 25 ºC in the light for 2 weeks (RT) or exposed to 37 ºC for 4 d and then transferred to 25 ºC for a further 10 d (HS). After treatments, fruits were visually classified as green (G), breaker (B), or colored (C). A representative result is shown in the picture. Quantification of the results from several independent experiments is shown in the graph.

Besides regulating DXS and PSY, ClpB3 and OR chaperones are known to improve tolerance to heat stress, possibly because they alleviate protein folding stress (Mishra and Grover 2016; Park *et al.*, 2016). To confirm further that increased levels of transcripts encoding SlClpB3, SlOR, and SlOR-like proteins led to increased chaperone activity and reduced protein folding stress in transgenic *ClpR1*-silenced fruit, we compared the tolerance to heat stress of MT and A94 fruit. Heat treatment of tomato MG fruit is known to inhibit several ripening-related processes, including chloroplast to chromoplast transition (Lurie *et al.*, 1996). Consistently, color change in MG fruit detached from MT plants and exposed to 37 ºC for 4 d was delayed compared with fruit incubated at 25 ºC (Fig. 7). The observed delay, however, was strongly attenuated in *ClpR1*-silenced A94 fruit. This result indicates that the up-regulation of chaperone-encoding genes detected in Clp protease-defective A94 fruits is likely to result in improved protein folding activity, indirectly improving protection of the fruit after exposure to heat stress.

### Multilevel regulation of fruit carotenoid metabolism by the Clp protease and plastidial ClpB3 and OR chaperones

Plastid proteases are required for the biogenesis and functioning of these essential organelles as they cleave and eliminate targeting peptides, degrade and recycle damaged, misfolded, and aggregated proteins, and maintain proper stoichiometry between different enzymes and pathways (Sakamoto, 2006; Nishimura *et al.*, 2016). Protein quality control (PQC) systems also include chaperones that promote correct protein folding and prevent the formation of non-functional and potentially deleterious protein aggregates. The work reported here provides evidence that specific plastidial PQC components play a central role for chromoplast differentiation and carotenoid accumulation during tomato fruit ripening.

The stromal Clp protease, which shows far more complexity in higher plants than in any other organism, has an essential role for chloroplast development that cannot be replaced by other proteases (Nishimura and van Wijk, 2015). However, the participation of this complex in chromoplast differentiation remained unexplored. Here we show that the tomato Clp protease influences carotenoid biosynthesis, turnover, and storage during fruit ripening. Our data indicate that defective Clp protease activity in tomato fruit during ripening results in major interferences of carotenoid composition as it impacts the expression of genes and accumulation of enzymes involved in carotenoid biosynthesis and degradation (Figs 4, 6) and disrupts the differentiation of chromoplast structures that influence their storage (Fig. 5). This activity appears to be connected to the expression of nuclear genes encoding plastid-targeted chaperones such as SlOR, SlOR-like, and SlClpB3, as defects in Clp protease function are signaled to the nucleus to induce their transcription (Fig. 6) and eventually mitigate protein folding stress (Fig. 7). Similarly, depletion of Clp protease activity in chloroplasts triggers changes in gene expression, resulting in increased abundance of chaperones to deal with the expected accumulation of misfolded and aggregated proteins and hence maintain plastid protein homeostasis (Ramundo *et al.*, 2014; Rochaix and Ramundo, 2015; Llamas *et al.*, 2017).

Under physiological conditions, the peak level of transcripts for most Clp complex subunits at the O stage (Fig. 2; Supplementary Table 1Supplementary Fig. S1) coincides with that for SlClpB3, SlOR, and SlOR-like chaperones as well as for the carotenoid biosynthetic enzymes DXS1 and PSY1 (Supplementary Table 1Supplementary Fig. S8). It is conceivable that increased Clp protease function during normal ripening might facilitate protein turnover and removal of proteins that are not further required or that lose their functionality as chloroplasts differentiate into chromoplasts. SlClpB3, SlOR, and SlOR-like chaperones might ensure optimal activity of DXS1 and PSY1 enzymes while preventing their removal when Clp protease activity is most actively working to support the differentiation of chromoplasts. It is likely that the co-ordinated activities of these plastidial chaperones and the Clp protease might further influence the folding and turnover of additional plastidial proteins whose activities can impact carotenoid accumulation. Proteomic studies in tomato have actually shown that the transition from chloroplasts to chromoplasts is associated with increased abundance of proteins involved in both the accumulation of carotenoids and the response to stress conditions causing protein misfolding (Barsan *et al.*, 2012; Suzuki *et al.*, 2015; Zeng *et al.*, 2015). In particular, the tomato small heat shock protein HSP21, a ripening-induced plastidial chaperone that protects against protein misfolding and aggregation, was found to contribute positively to the conversion of fruit chloroplasts to chromoplasts and the accumulation of carotenoids during normal fruit development (Neta-Sharir *et al.*, 2005). The nuclear gene encoding HSP21 in Arabidopsis is also induced by the retrograde pathway that acts in response to reduced Clp protease activity in chloroplasts and up-regulates *ClpB3* expression (Llamas *et al.*, 2017). Together, plastidial PQC systems might be up-regulated during tomato fruit ripening to deal with proteome changes and protein folding stress resulting from the transformation of chloroplasts into chromoplasts.

We propose that Clp protease-regulated expression of chaperone-encoding genes might be a compensatory mechanism triggered when Clp protease activity becomes compromised. If Clp protease activity decreases too much during ripening (as in the case of A94 fruit), the expected protein turnover defects would result in (i) incomplete differentiation of chromoplasts (Fig. 5) and (ii) accumulation of non-functional proteins, hence triggering the retrograde signal up-regulating *SlClpB3*, *SlOR*, and *SlOR-like* genes (Fig. 6) eventually to mitigate protein folding stress (Fig. 7). A close connection between Clp protease and OR activities could also synergistically contribute to the most obvious phenotypic effect of *ClpR1*-silenced lines, namely the orange color of ripe fruit (Fig. 3). Maintenance of similar levels of lutein and β-carotene in R fruit compared with O fruit of transgenic lines (Fig. 4A) might result from incomplete disintegration of residual thylakoid membranes rich in these two carotenoids (Fig. 5) or from altered balance between rates of synthesis (via DXS1, PSY1, LCYB, CYCB, and CYP97C11) and degradation (via CCDs) (Figs 4B, 6). However, up-regulated expression of *SlOR* and *SlOR-like* genes resulting in increased OR activity (Fig 6) could further explain the phenotype of Clp-defective fruit. Recent results suggest that inhibited degradation of carotenoids (particularly β-carotene and lutein) is a pivotal part of the mechanism by which OR proteins promote fruit chromoplast differentiation (Chayut *et al.*, 2017). Therefore, increased levels of OR chaperones in ripe A94 tomatoes could contribute to their enrichment in lutein and β-carotene (Fig. 4), but also their altered chromoplast ultrastructure (Fig. 5), and even their higher levels of PSY protein (Fig. 6) (Zhou *et al.*, 2015; Park *et al.*, 2016; Chayut *et al.*, 2017). A better understanding of how these PQC networks of proteases (Clp complex) and chaperones (OR and ClpB3) act together to ensure appropriate accumulation of carotenoids during tomato fruit ripening should contribute to a more rational engineering of carotenoid-enriched (i.e. healthier) fruits and vegetables.

## Supplementary data

Supplementary data are available at *JXB* online.

Fig. S1. Clp protease subunits and their expression during tomato fruit ripening.

Fig. S2. HPLC chromatograms of carotenoids in ripe fruit.

Fig. S3. Alignment of the region harboring the catalytic triad of Clp proteolytic subunits.

Fig. S4. Position and validation of amiRNA sequences.

Fig. S5. Transcript levels of ripening marker genes in wild-type and *ClpR1*-silenced fruits.

Fig. S6. Ripening rate of transgenic tomato lines.

Fig. S7. Alignment of OR proteins from several plants.

Fig. S8. Expression pattern of genes encoding carotenoid biosynthetic enzymes and chaperones controlling their stability during tomato fruit ripening.

Table S1. Carotenoid-related proteins.

Table S2. Tomato homologs of the Clp protease complex subunits.

Table S3. Primers used in this work.

Table S4. Comparison of reference genes for qPCR analysis of carotenoid-related gene expression during tomato fruit ripening.

supplementary_figures_S1_S8_tables_S1_S4
