# Supplementary material for: Interference with Clp protease impairs carotenoid accumulation during tomato fruit ripening
Source: J Exp Bot. 2018 Jan 29;69(7):1557–68. doi: 10.1093/jxb/erx491 (PMC5888976; doi:10.1093/jxb/erx491)
Supplement: supplementary_figures_S1_S8_tables_S1_S4 [file erx491_suppl_supplementary_figures_s1_s8_tables_s1_s4.pdf]

Supplementary Figures

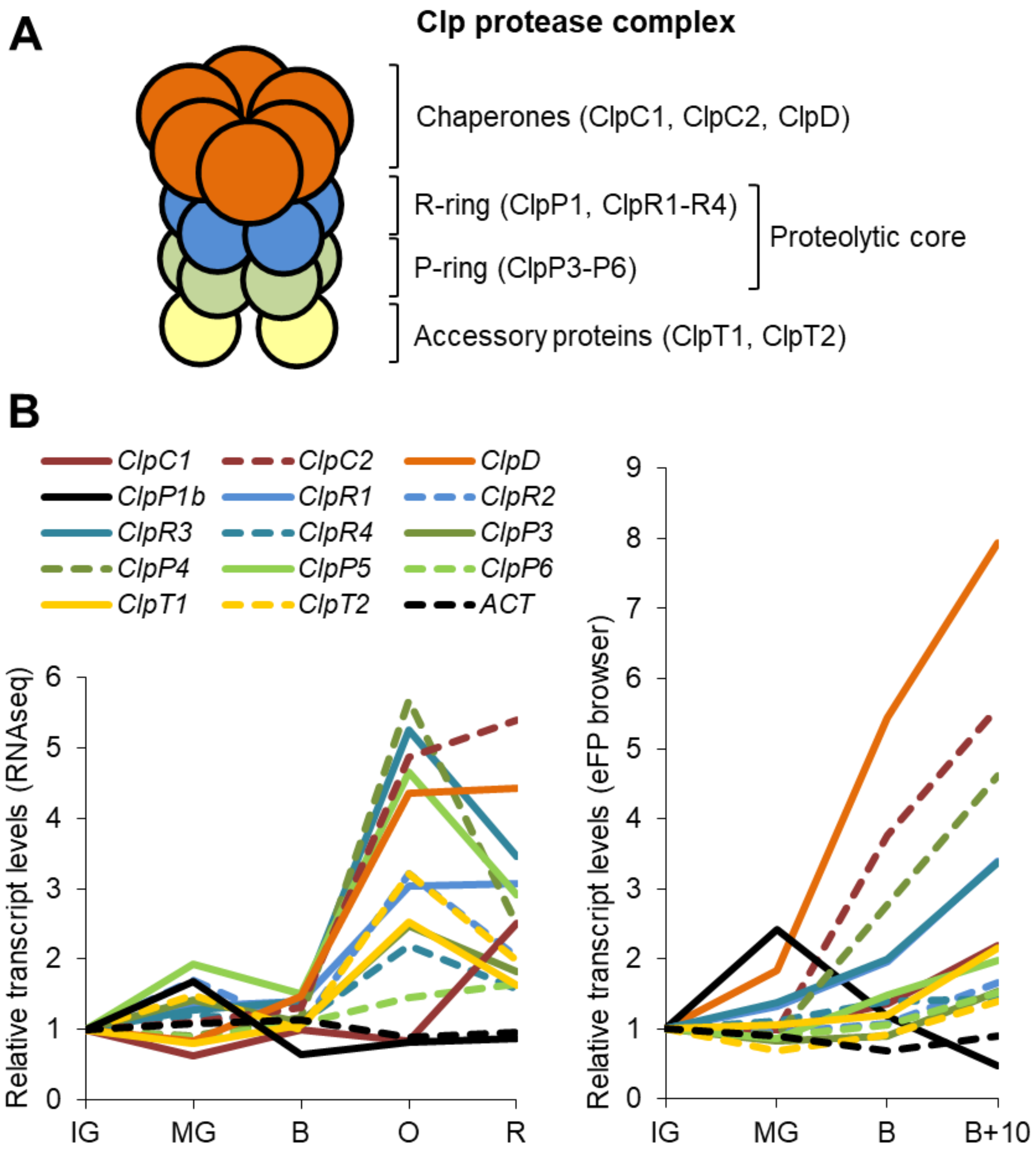

Fig. S1. Clp protease subunits and their expression during tomato fruit ripening. (A) The cartoon shows the structural organization of the stromal Clp complex. Chaperones unfold and translocate protein substrates into the proteolytic chamber for degradation into small peptides that are emitted through the lateral pores of the chamber. (B) Graphs show the level of transcripts for Clp subunits in the pericarp (flesh) of tomato fruit. The profile of the qPCR reference gene *ACT* (Solyc04g011500) is also shown. Graph in the left represents RNAseq data from the Aharoni lab (www.weizmann.ac.il/plants/aharoni/made-aa-lab) corresponding to immature green (IG), mature green (MG), breaker (B), orange (O), and red ripe (R) fruit. Graph in the right contains data from the tomato eFP browser (http://bar.utoronto.ca/efp_tomato) corresponding to fruit at the following stages: 3 cm (IG), MG, breaker (B), and 10 days after breaker (B+10). In both graphs, results are represented relative to the levels in IG fruit. See Table S2 for accessions.

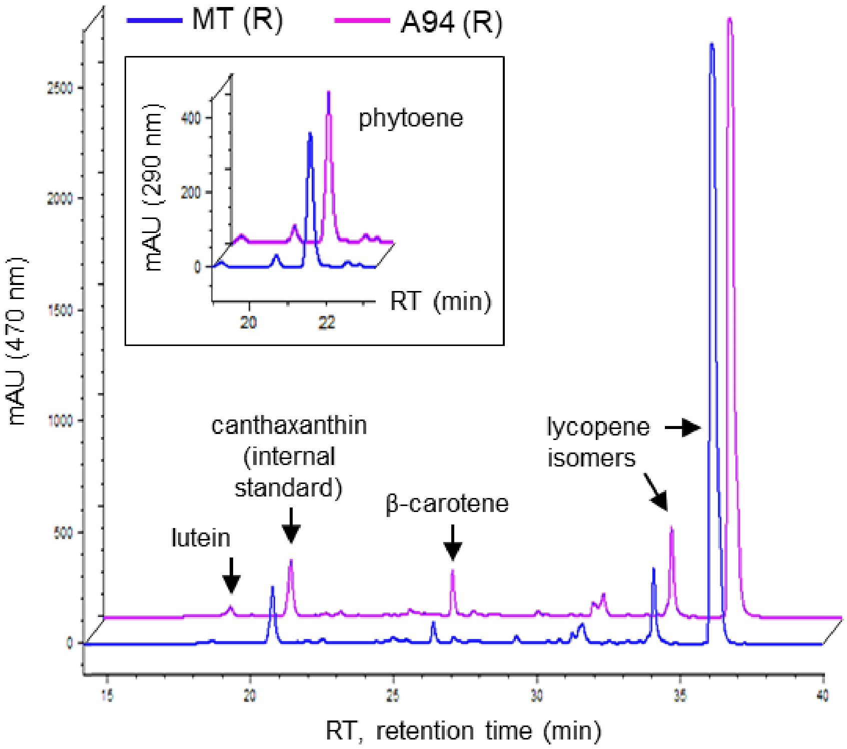

Fig. S2. HPLC chromatograms of carotenoids in ripe fruit. Pericarp from fruit collected from MT and A94 plants at the R stage (52 DPA) was used for carotenoid extraction and HPLC analysis using the non-plant carotenoid canthaxanthin as an internal standard as described (Rodriguez-Concepcion *et al.*, 2004). Representative HPLC chromatograms are shown. Lutein, β-carotene, and lycopene, together with canthaxanthin, were detected at 470 nm. Phytoene was detected at 290 nm (inset).

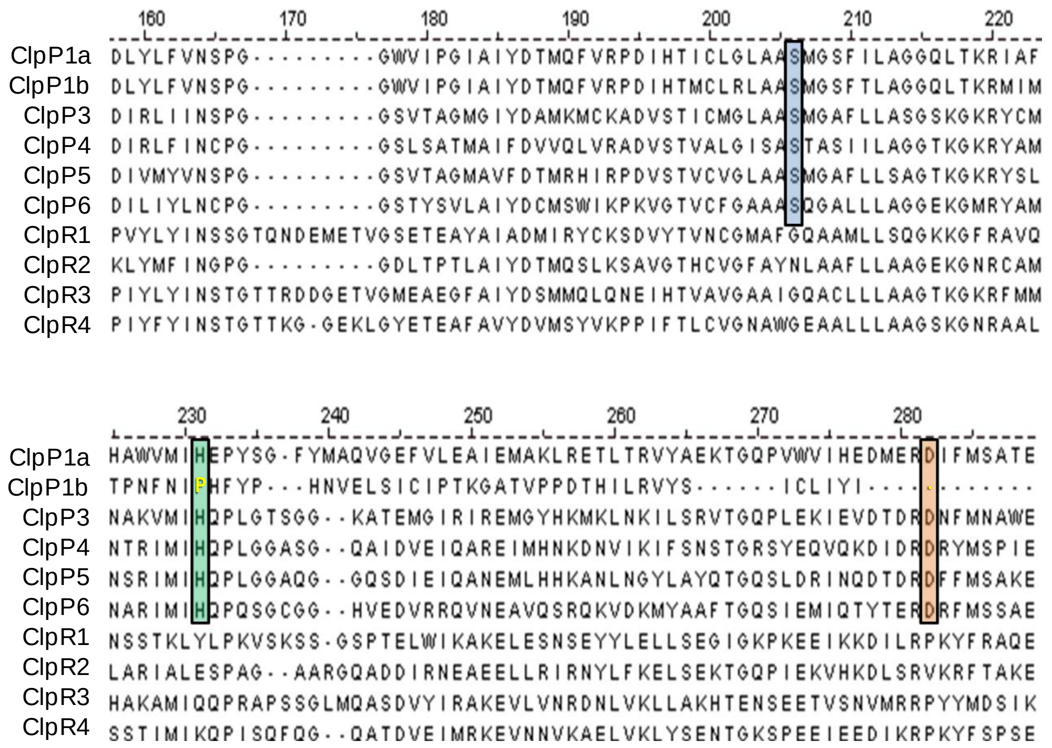

Fig. S3. Alignment of the region harboring the catalytic triad of Clp proteolytic subunits. Multiple alignment was performed with the MEGA6 software using the protein sequences listed in Table S2. Triad residues are boxed.

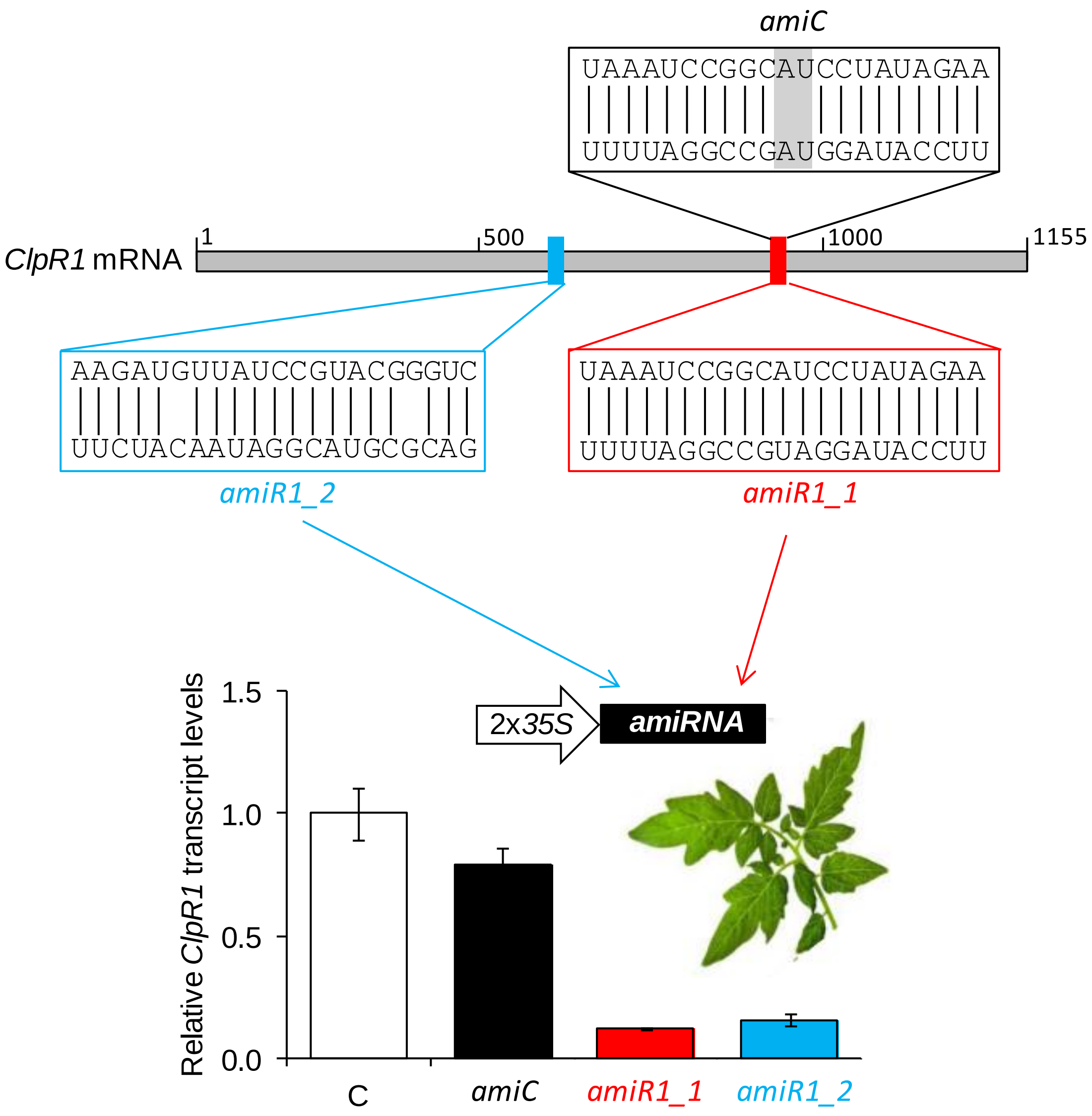

Fig. S4. Position and validation of amiRNA sequences. The upper cartoon shows the regions in the tomato *ClpR1* mRNA targeted by *amiR1_1* (red box) and *amiR1_2* (blue box). The mutations in the *amiR1_1* sequence that render the construct inactive (*amiC*, black box) are highlighted in gray. For validation, amiRNAs were cloned under the control of the *2x35S* promoter and the generated constructs were agroinfiltrated in independent tomato leaves. The lower graph shows a qPCR analysis of *ClpR1* transcript levels in leaves transiently expressing the indicated amiRNAs. Data correspond to mean ± SEM of n=3 leaves and they are represented relative to *ClpR1* transcript levels in leaves agroinfiltrated with a similar vector harboring an unrelated amiRNA (C).

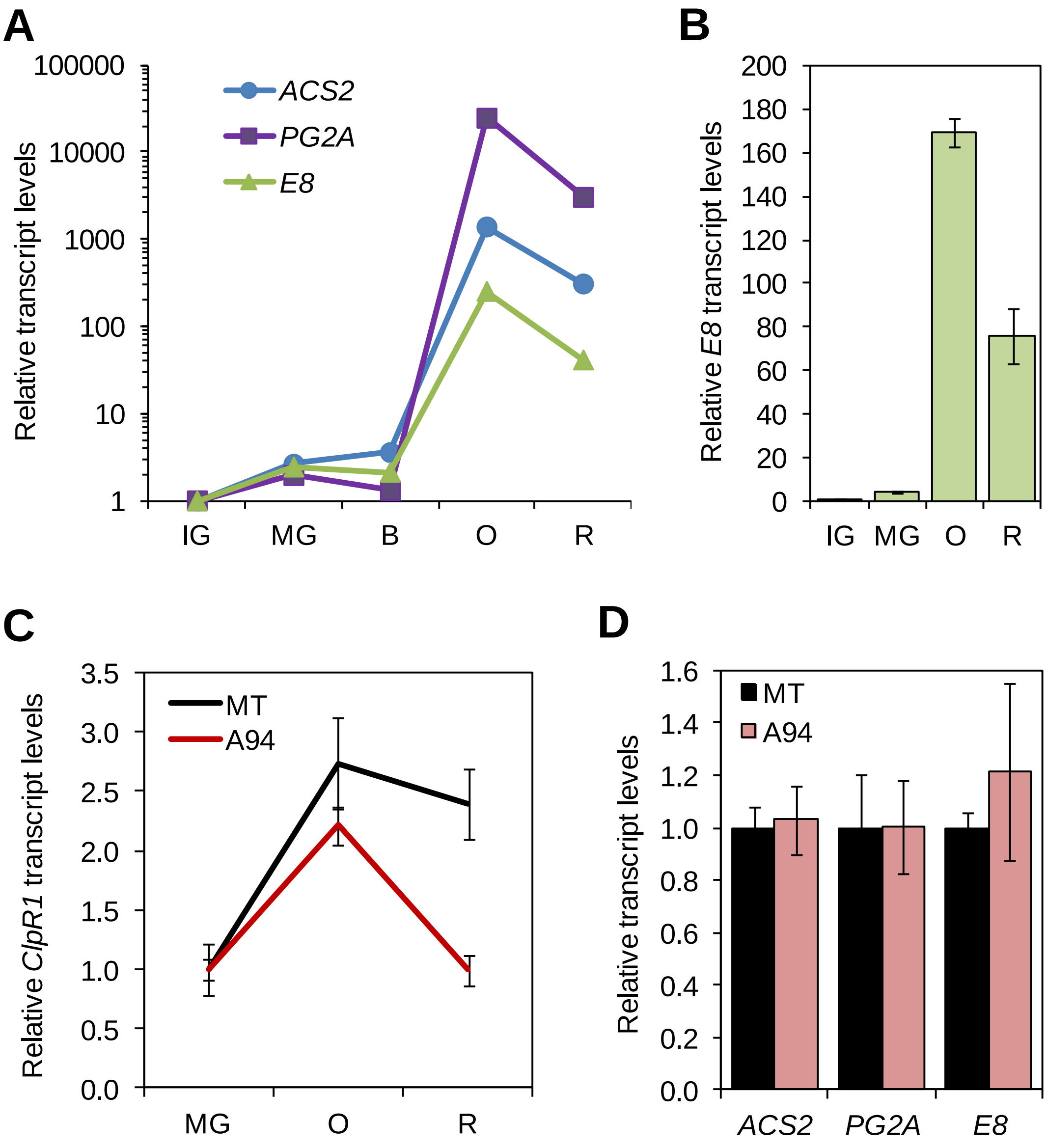

Fig. S5. Transcript levels of ripening marker genes in wild-type and *ClpR1*-silenced fruits. (A) Level of transcripts of ripening-related genes *E8* (Solyc09g089580), *PG2A* (Solyc10g080210) and *ACS2* (Solyc01g095080) in the pericarp (flesh) of tomato fruit at the indicated stages of ripening (RNAseq data from the Aharoni lab represented in a log10 scale relative to the levels in IG fruit; see Fig. S1). (B) Quantitative RT-PCR (qPCR) analysis of *E8* transcripts in MT fruits. Mean ± SEM of n≥3 fruits are shown relative to IG samples. (C) Transcript levels of *ClpR1* in fruits from MT and *E8:amiR1* (line A94) plants at the indicated stages. Mean ± SEM of n≥3 fruits are shown relative to MT fruit at the MG stage. No statistically significant differences relative to reference MT samples were found. (D) Levels of *E8*, *PG2A* and *ACS2* transcripts detected by qPCR in ripe fruits from MT and A94 plants. Mean ± SEM of n≥3 fruits are shown relative to MT samples. No statistically significant differences relative to reference MT samples were found.

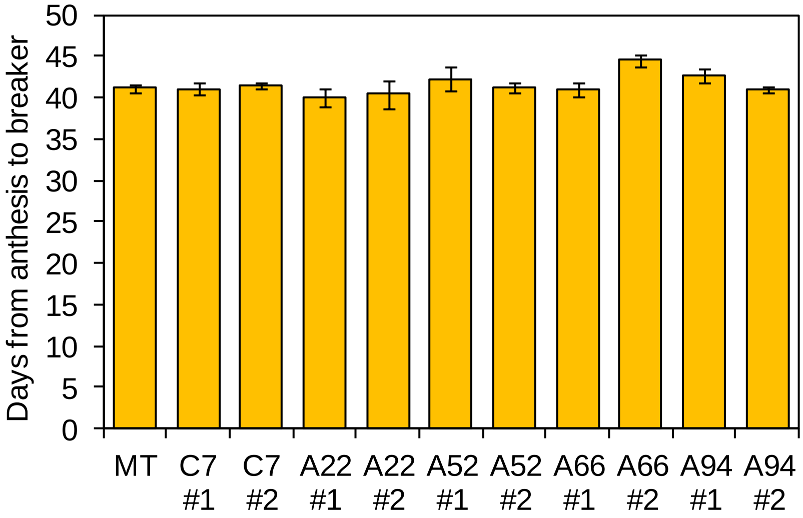

Fig. S6. Ripening rate of transgenic tomato lines. MT and transgenic plants of the indicated lines were grown in the greenhouse. At least five flowers from each of two plants of every transgenic line (#1 and #2) were tagged at anthesis and left to self-pollinate. The graph represents the mean and SEM of the number of days from the day of anthesis (day 0) to the day when the first signs of chlorophyll loss and carotenoid overaccumulation were visually detected (breaker stage). No statistical differences were found.

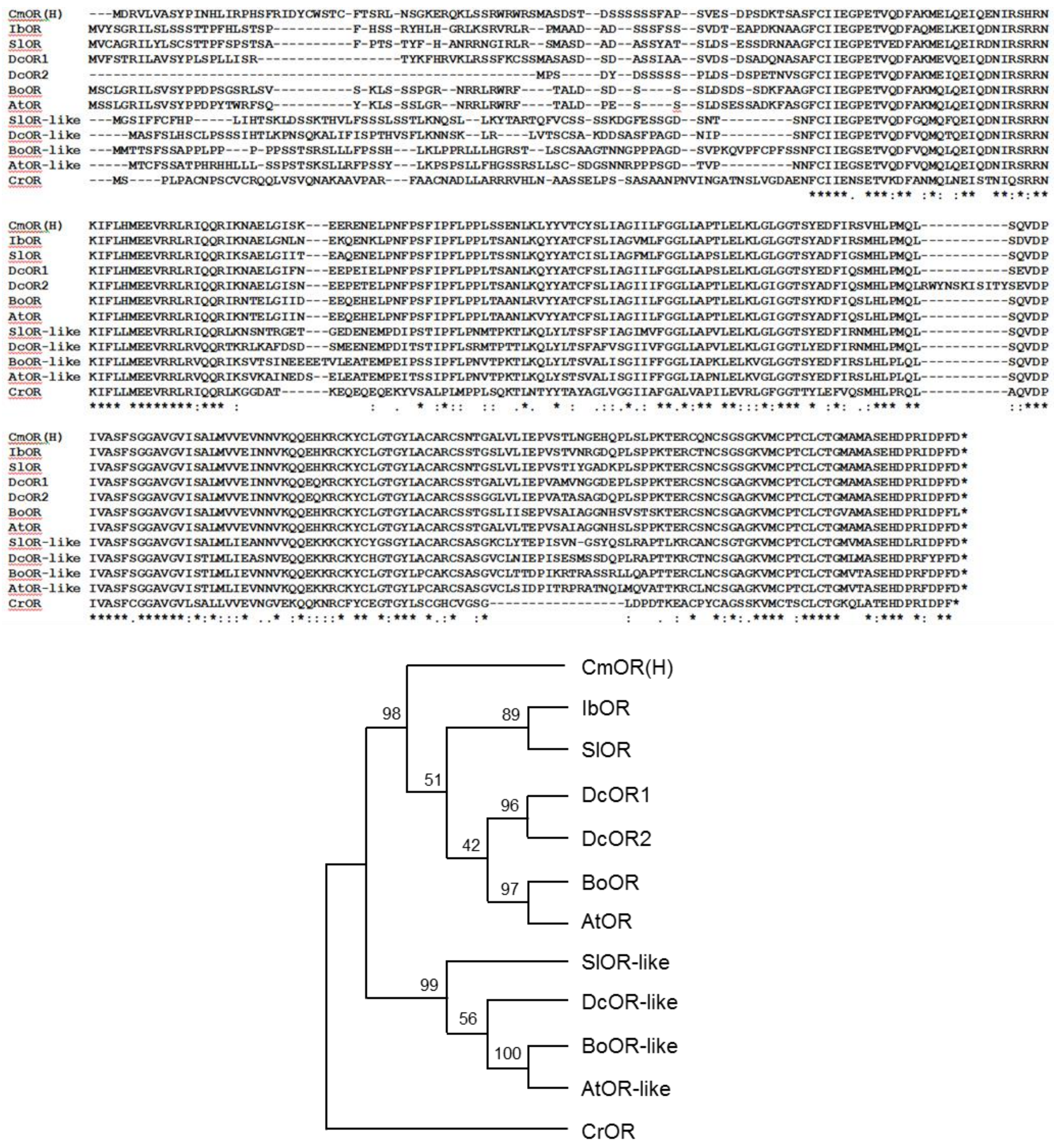

Fig. S7. Alignment of OR proteins from several plants. Multiple alignment was performed with the MEGA6 software using the following protein sequences: *Chlamydomonas reinhardtii* CrOR (Cre06.g279500.t1.1), *Cucumis melo* CmOR(H) (A0A0D3MU50.1), *Arabidopsis thaliana* AtOR (AT5G61670.1) and AtOR-like (AT5G06130.2), *Brassica oleracea* BoOR (Bol036294) and BoOR-like (Bol024498), *Daucus carota* DcOR1 (DCAR_020166), DcOR2 (DCAR_009463), and DcOR-like (DCAR_009172), *Ipomoea batatas* IbOR (HQ828087), and *Solanum lycopersicum* SlOR (Solyc03g093830.2.1) and SlOR-like (Solyc09g010110). A Maximum Likelihood tree constructed with these data is also shown.

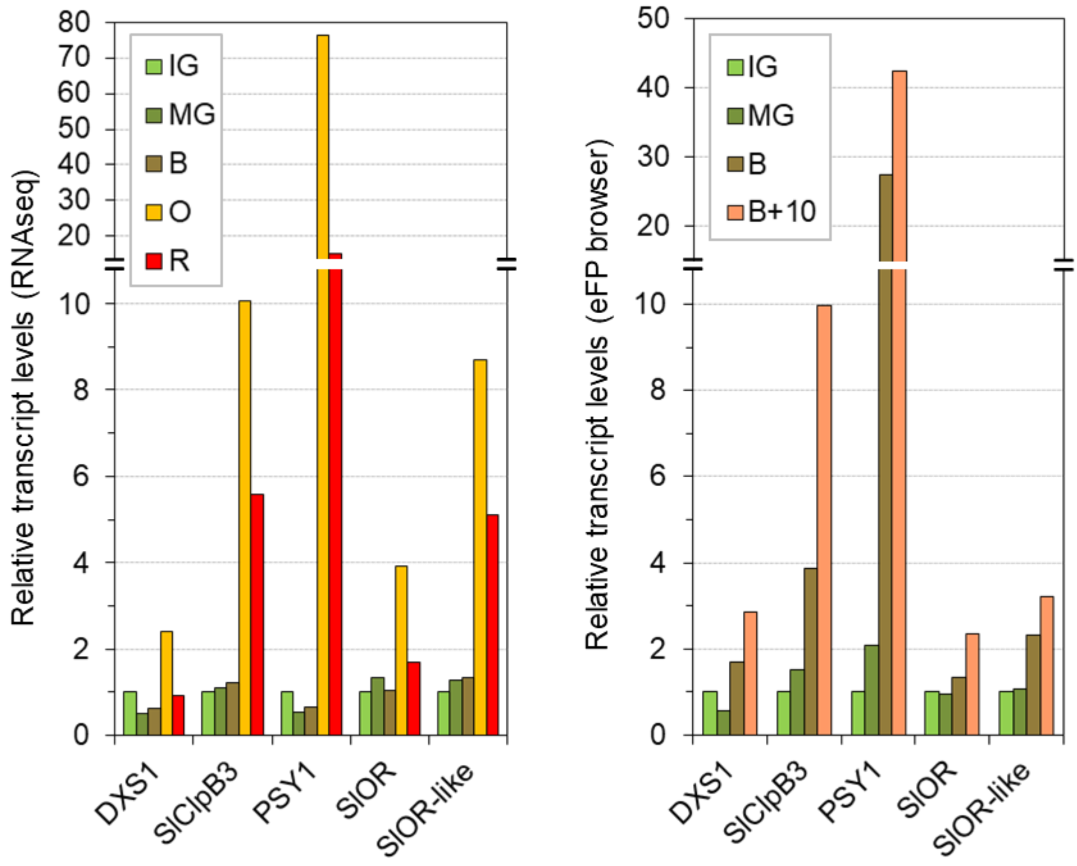

Fig. S8. Expression pattern of genes encoding carotenoid biosynthetic enzymes and chaperones controlling their stability during tomato fruit ripening. Data correspond to publicly available RNAseq results (left graph) and eFP browser data (right graph) from tomato pericarp samples as described in Fig. S1. Values are represented in a log10 scale relative to the levels in IG fruit. Accessions are listed in Table S1.

Supplementary Tables

Table S1. Carotenoid-related proteins.

	Arabidopsis		Tomato	
**Name**	**Protein abbreviation**	**Gene identifier**	**Protein abbreviation**	**Gene identifier**
deoxyxylulose-5–phosphate synthase	DXS	At4g15560	DXS1	Solyc01g067890
plastidial Hsp100 type B chaperone	ClpB3	At5g15450	SlClpB3	Solyc02g088610
phytoene synthase	PSY	At5g17230	PSY1	Solyc03g031860
phytoene desaturase	PDS	At4g14210	PDS	Solyc03g123760
ζ-carotene desaturase	ZDS	At3g04870	ZDS	Solyc01g097810
15-*cis* -ζ-carotene isomerase	Z-ISO	At1g10830	Z-ISO	Solyc12g098710
carotenoid isomerase	CRTISO	At1g06820	CRTISO	Solyc10g081650
lycopene β–cyclase	LCYB	At3g10230	LCYB	Solyc10g079480
			CYCB	Solyc06g074240
lycopene ε–cyclase	LCYE	At5g57030	LCYE	Solyc12g008980
non-heme di-iron carotenoid hydroxylase	BCH1	At4g25700	BCH1	Solyc06g036260
	BCH2	At5g52570	BCH2	Solyc03g007960
cytochrome P450 carotenoid hydroxylase	CYP97A3	At1g31800	CYP97A29	Solyc04g051190
	CYP97B3	At4g15110	CYP97B3	Solyc05g016330
	CYP97C1	At3g53130	CYP97C11	Solyc10g083790
carotenoid cleavage dioxygenase	CCD1	At3g63520	CCD1A	Solyc01g087250
			CCD1B	Solyc01g087260
	CCD4	At4g19170	CCD4A	Solyc08g075480
			CCD4B	Solyc08g075490
	CCD7	At2g44990	CCD7	Solyc01g090660
	CCD8	At4g32810	CCD8	Solyc08g066650
orange chaperone	AtOR	At5g61670	SlOR	Solyc03g093830
	AtOR-like	At5g06130	SlOR-like	Solyc09g010110

Table S2. Tomato homologues of the Clp protease complex subunits.

Protein abbreviation	Arabidopsis gene identifier	Tomato gene identifier	Identity (%)[2]	Subcellular localization[3]
ClpP1	AtCg00670	emb|AM087200[1]	75	P
		Solyc01g007490[1]	75	other
		Solyc09g065790	71	other
ClpP2	At5g23140	Solyc04g009310	85	M
ClpP3	At1g66670	Solyc02g091280	75	P
ClpP4	At5g45390	Solyc08g075750	64	P
ClpP5	At1g02560	Solyc01g100520	78	P
ClpP6	At1g11750	Solyc10g051310	70	P
ClpR1	At1g49970	Solyc10g049710	66	P
ClpR2	At1g12410	Solyc08g079620	68	P
ClpR3	At1g09130	Solyc01g099690	77	P
ClpR4	At4g17040	Solyc08g077890	76	P
ClpC1	At5g50920	Solyc12g042060	90	P
ClpC2	At3g48870	Solyc03g118340	86	P
ClpD	At5g51070	Solyc03g117950	67	P
ClpT1	At4g25370	Solyc03g007110	56	P
ClpT2	At4g12060	Solyc08g079660	47	P

[1] Same sequence

[2] Compared to the corresponding Arabidopsis protein sequence

[3] TargetP (P, plastid; M, mitochondria)

Table S3. Primers used in this work.

Use	Name	Sequence (5'-3')
amiRNA constructs	miR1-A	GGGGACAACTTTTCTATACAAAGTTGCTCCCCAAACACACGCTCGGA
	miR1-B	GGGGACAACTTTATTATACAAAGTTGTCCCCATGGCGATGCCTTAA
	R1_1 I-s	GATTTTAGGCCGATGGATACCTTCTCTCTTTTGTATTCCA
	R1_1 II-a	AGAAGGTATCCATCGGCCTAAAATCAAAGAGAATCAATGA
	R1_1 III*s	AGAAAGTATCCATCGCCCTAAATTCACAGGTCGTGATATG
	R1_1 IV*a	GAATTTAGGGCGATGGATACTTTCTACATATATATTCCTA
	R1_2 I-s	GATTCTACAATAGGCATGCGCAGCTCTCTTTTGTATTCCA
	R1_2 II-a	AGCTGCGCATGCCTATTGTAGAATCAAAGAGAATCAATGA
	R1_2 III*s	AGCTACGCATGCCTAATGTAGATTCACAGGTCGTGATATG
	R1_2 IV*a	GAATCTACATTAGGCATGCGTAGCTACATATATATTCCTA
	C I-s	GATTTTAGGCCGTAGGATACCTTCTCTCTTTTGTATTCCA
	C II-a	AGAAGGTATCCTACGGCCTAAAATCAAAGAGAATCAATGA
	C III*s	AGAAAGTATCCTACGCCCTAAATTCACAGGTCGTGATATG
	C IV*a	GAATTTAGGGCGTAGGATACTTTCTACATATATATTCCTA
RT-qPCR	ACT-F	CCTTCCACATGCCATTCTCC
	ACT-R	CCACGCTCGGTCAGGATCT
	ClpR1-F	CCACTTTCTTGCCCTACTC
	ClpR1-R	GAAGAGAATCTGAAAAGAAG
	ClpR2-F	CACTGCTAAAGAAGCTCTTG
	ClpR2-R	CAGTGATATCCCTCGGCG
	ClpR3-F	TTCTTTTCAAGCTTCCGTTGA
	ClpR3-R	CAACCGCAAGCACGTGGC
	ClpR4-F	ATGGAAGCTGTCACTATTGC
	ClpR4-R	TGAGGCACGGCAACTCGC
	ClpP3-F	TTGGTTGATGCTGTTATAGATGAC
	ClpP3-R	TTTTGGTGGAGGTGCATCCT
	ClpP4-F	ATTGACGGTGTAATTGACAGAGA
	ClpP4-R	ATTTCATAGGGTCTTGGATCAAA
	ClpP5-F	TCATGAGCGCAAAGGAAGCT
	ClpP5-R	CAAGTGGTTGAAGGGCTTTCA
	ClpP6-F	GTTCAATGAGTACGAATCCGG
	ClpP6-R	GGCATGATGGGCGGATTAG
	ClpB3-F	TCGACCGGGTAAATCTTGAG
	ClpB3-R	TGACGCTGCAAGGTGTTTAG
	DXS1-F	TGACCATGGATCTCCTGTTG
	DXS1-R	GCCTCTCTGGTTTGTCCAAG
	PSY1-F	GCCATTGTTGAAAGAGAGGGTG
	PSY1-R	AGGCAAACCAACTTTTCCTCAC
	LCYE-F	GCCACAAGAACGAAAACGAC
	LCYE-R	CGCGGAAAAATGACCTTATC
	LCYB-F	TTGTGGCCCATAGAAAGGAG
	LCYB-R	GGCATCGAAAAACCTTCTTG
	CYCB-F	TGGCAAGGGTTCCTTTCTTC
	CYCB-R	AGTCATGTTTGAGCCATGTCC
	BCH1-F	TGCTGCTGTAGGAATGGAGTT
	BCH1-R	TCCTTCTCTTGGTTTGTGGTG
	BCH2-F	CTCGGACAAATTTGATGGTGT
	BCH2-R	CCTTCGGTTGACTTCCTTTTC
	CYP97A29-F	TTGGGAAGAAGCCGATAGATT
	CYP97A29-R	ACCGAAGGGAAGGTAACTGAA
	CYP97B3-F	AATGAAGAAAGCGCAGTCAGA
	CYP97B3-R	CCACGACAATAAGTCGCAAAT
	CYP97C11-F	GCTGTGGTTCCATCTCTTCAC
	CYP97C11-R	GCCAGAAATTGCATCAGGTAA
	CCD1A-F	TTGATTACCTGCCGCCTTGT
	CCD1A-R	CATATAGCTCATTGCAGAAATTC
	CCD1B-F	AGAATCCAGATCTTGACGCGATT
	CCD1B-R	CCTCATCTCATACAACTCATTTG
	CCD4A-F	GTGGGGTAGTGAGTAGACATCC
	CCD4A-R	ACGTCCTACATGCCACTATGC
	CCD4B-F	GGAATGGTGAGCAGACATCC
	CCD4B-R	GACGATCAATTTCTGCTACGG
	CCD7-F	TCAGTCCAAAAGTGGTCAGC
	CCD7-R	TGCATATTCAACCACAAGAAGG
	CCD8-F	CCTTCTGAACCATTCTTTGTGG
	CCD8-R	AACCCAACAACCATGTAGCC
	OR-F	TTGGGACTAGGAGGCACATC
	OR-R	AGAGATCACCCCAACTGCAC
	OR-I-F	GGATTATGGTCTTCGGTGGA
	OR-I-R	ACCTGGCTCAATTGCATAGG
	ACS2-F	CGTTTGAATGTCAAGAGCCAGG
	ACS-R	TCGCGAGCGCAATATCAAC
	PG2A-F	ATGGCAATGGACAAGTATGGTG
	PG2A-R	TTAAGGCCGTTGGTGCATC
	E8-F	AGCTGCAAGTTGGAGAGACACG
	E8-R	CCGCATGGAGTTGGAAATTC

Table S4. Comparison of reference genes for qPCR analysis of carotenoid-related gene expression during tomato fruit ripening.

Gene type	Gene name	Gene identifier	Primer efficiency	Expression level at MG / B / B+10 [(3)]
**Reference**	*CAC*	Solyc08g006960	0.88[(1)] - 0.93[(2)]	24.28 / 21.75 / 25.65
	EXP	Solyc07g025390	0.88[(1)] - 0.87[(2)]	13.40 / 8.05 / 14.91
	ACT	Solyc04g011500	0.96	163.12 / 124.90 / 162.10
**Carotenoid-related**	*ClpR1*	Solyc10g049710	0.99	132.75 / 197.65 / 341.93
	DXS1	Solyc01g067890	0.90	59.54 / 176.03 / 295.39
	PSY1	Solyc03g031860	0.91	188.53 / 2466.46 / 3813.63

[1] Gonzalez-Aguilera *et al.* , 2016

[2] Exposito-Rodriguez *et al.* , 2008

[3] Tomato eFP browser values at the indicated ripening stages (see Fig. S1 and S8).

References

Expósito-Rodríguez M, Borges AA, Borges-Pérez A, Pérez JA. 2008. Selection of internal control genes for quantitative real-time RT-PCR studies during tomato development process. BMC Plant Biology **8**, 131.

González-Aguilera KL, Saad CF, Chávez Montes RA, Alves-Ferreira M, de Folter S. 2016. Selection of Reference Genes for Quantitative Real-Time RT-PCR Studies in Tomato Fruit of the Genotype MT-Rg1. Frontiers in Plant Science **7**, 1386.

Rodríguez-Concepción M, Forés O, Martínez-García JF, González V, Phillips MA, Ferrer A, Boronat A. 2004. Distinct Light-Mediated Pathways Regulate the Biosynthesis and Exchange of Isoprenoid Precursors during Arabidopsis Seedling Development. Plant Cell **16**, 144-156.
